# Preparation of P‐Doped Ni Catalyst Using Deep Eutectic Solvents and Its Excellent Hydrogen Evolution Performance in Water Splitting

**DOI:** 10.1002/open.202500023

**Published:** 2025-04-10

**Authors:** Chenyun Zhang, Jiahao Wang

**Affiliations:** ^1^ School of Ceramics Wuxi Vocational Institute of Arts & Technology Yixing 214200 China

**Keywords:** deep eutectic solvent, hydrogen evolution reaction, phosphorus‐doped nickel catalyst, tetraalkylphosphonium chloride([P_4444_]Cl)/glycerol, water splitting

## Abstract

A deep eutectic solvent (DES) was prepared by mixing tetraalkylphosphonium chloride ([P_4444_]Cl) and glycerol in a 1:2 molar ratio. Nickel nitrate was dissolved in this DES, and then the mixture was heated at 350 °C to obtain phosphorus‐doped nickel catalysts. In this system, the DES plays multiple crucial roles. (1) It serves as a reaction medium, providing a suitable solvent environment for the synthesis of Ni‐based catalysts. (2) It actively participates in the catalyst preparation as a reactant. [P_4444_]Cl acts as a P source, while glycerol effectively promotes the formation of nickel metal as a reducing agent. (3) It exhibits a templating effect, inducing the morphology of product to be a unique thin sheet with angular edges. The synthesized P‐doped Ni material exhibits excellent hydrogen evolution performance in water splitting. In an acidic environment, when the current densities reached 10 and 20 mA cm^−2^, the overpotentials were only 90.2 and 115.9 mV, with a Tafel slope as low as 55.6 mV dec^−1^. In an alkaline environment, the P‐doped Ni also performed well. The *η*_10_ and *η*_20_ values were 107.0 and 145.4 mV, respectively. The Tafel slope was 57.0 mV dec^−1^.

## Graphical abstract

A deep eutectic solvent (DES) prepared by mixing tetraalkylphosphonium chloride ([_P4444_]Cl) and glycerol is applied in the synthesis of P‐doped Ni materials. The DES not only serves as the reaction medium but also participates in the catalyst preparation. The synthesized P‐doped Ni catalyst exhibits excellent performance in HER in both acidic and alkaline environments.

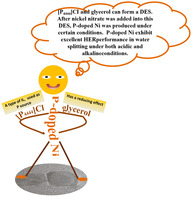

## 1 Introduction

With the continuous growth of global energy demand and the increasingly severe environmental issues, the development of efficient and sustainable energy conversion and storage technologies has become particularly crucial. Among various energy sources, hydrogen is considered the most potential renewable and clean energy source to replace fossil fuels in the future due to its abundant reserves, extremely high calorific value, and no carbon dioxide emissions after combustion.^[^ ^1^, ^2^ ^]^ Electrolytic water splitting is considered as one of the most promising routes to produce H_2_.^[^ ^3^, ^4^ ^]^ However, the rate and efficiency of the hydrogen evolution reaction (HER) during this process largely depend on catalysts. Therefore, the study on the HER catalysts is one of the current research hotspots.^[^ ^5^ ^]^

Nickel‐based materials have emerged as a promising class of catalysts for water splitting due to their relatively low cost, good stability, and moderate catalytic activity.^[^ ^6^, ^7^ ^]^ During the process of water splitting, water molecules initially adsorb onto the surface of catalyst. The outer electrons of Ni atoms interact suitably with O atoms in H_2_O, effectively activating the water molecules. Additionally, nickel exhibits a well‐organized crystalline structure, which is characterized by appropriate atomic spacing and arrangement within its lattice. This arrangement offers sufficient space for the adsorbed hydrogen atoms (H*), reaction intermediates, allowing them to stably adsorb onto its surface and efficiently combine to form H_2_ at favorable potentials.^[^ ^8^ ^]^ However, Ni‐based catalysts still require further optimization to meet the demands of hydrogen production. Therefore, researchers have attempted various methods, such as morphology control, surface modification, heteroatom doping, and so on. One effective strategy to enhance the catalytic properties is through doping with phosphorus, which can modify the electronic structure and surface chemistry of the catalyst, leading to improved activity and stability.^[^ ^9^, ^10^ ^]^

In recent years, the utilization of deep eutectic solvents (DESs) in the preparation of transition metal catalysts has increasingly garnered significant attention and interest.^[^ ^11^, ^12^, ^13^ ^]^ DESs, as a novel type of environmentally friendly solvent, possess several distinct advantages over traditional solvents. They exhibit excellent solubility, low toxicity, and are environmentally friendly. Importantly, DESs can be tailored through the design of their composition and ratio of hydrogen‐bond donors (HBDs) and hydrogen‐bond acceptors (HBAs). The unique chemical environment of DESs allows them to serve multiple roles: acting as templating agents to control the morphology of catalysts and as reactive components that directly participate in the catalytic reaction. These functions render DESs highly appealing for the synthesis of inorganic catalysts and provide a more economical alternative for chemical synthesis, material extraction, catalytic systems, and the energy sector compared to other solvents.^[^ ^14^, ^15^, ^16^ ^]^ A large number of reports have reported the preparation of nickel‐based catalysts using DESs.^[^ ^17^, ^18^, ^19^ ^]^

This study aims to explore a novel synthesis method for P‐doped Ni catalysts with excellent HER performance in water splitting. To this end, a DES composed of tetrabutylphosphonium chloride ([P_4444_]Cl) and glycerol was designed, and Ni(NO_3_)_2_ was dissolved in it. Annealing this mixture under N_2_ protection yielded P‐doped nickel catalysts with angular, lamellar structures. Notably, the synthesized P‐doped Ni material exhibited excellent HER performance in water splitting, with low overpotentials and a favorable Tafel slope in both acidic and alkaline environments.

## 2 Results and Discussion

### 2.1 Preparation and Characterization of Nickel‐Based Catalysts

#### 2.1.1 Preparation and Characterization of Ni‐DES

The DES was successfully prepared by mixing [P_4444_]Cl and glycerol in a 1:2 molar ratio. Then Ni(NO_3_)_2_·6H_2_O was dissolved in the prepared DES and annealed in a tube furnace at 350 °C for 3 h. The obtained black powder was labeled as Ni‐DES. DES provides a stable solvent environment for the synthesis of Ni‐DES.

**Figure 1:**
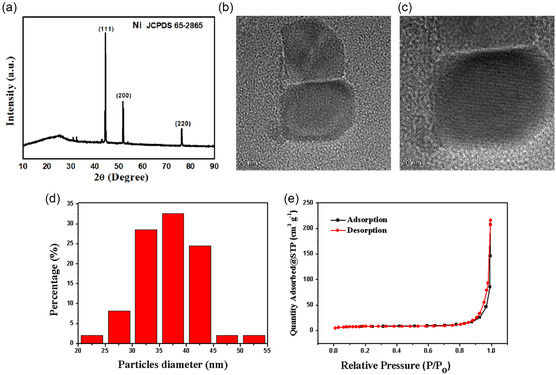
a) XRD pattern, b,c) the typical low‐ and high‐magnification TEM images, d) the particle size distribution, and e) the nitrogen adsorption isotherm of Ni‐DES.

The crystalline phase structure of the synthesized product was characterized by X‐ray diffraction (XRD) patterns. The major diffraction peaks appeared at ≈44.4°, 51.8°, and 76.0°, corresponding to the (111), (200), and (220) crystal planes, respectively. All diffraction peaks matched well with nickel (JCPDS 65‐2865) (**Figure** **1**a). The XRD characterization results showed that the synthesized Ni‐DES exhibited good crystallinity. Figure 1b,c show the typical low‐ and high‐magnification transmission electron microscopy (TEM) images of the as‐prepared Ni‐DES. Thin platelet with the angular can be clearly observed, and a particle size was measured at 35.71 ± 6.93 nm (Figure 1d). This thin platelet morphology has a large surface area energy, providing more active sites to adsorb more reactant molecules. This indicated that the DES played an effective templating role during the synthesis process, precisely regulating the morphology of product. The nitrogen adsorption isotherm indicated that the Ni‐DES possessed a high specific surface area of 23.63 m^3^ g^−1^ (Figure 1e).

**Figure 2:**
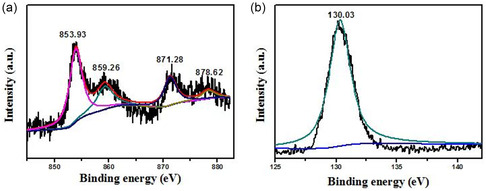
a) XPS spectra of the Ni 2*p* and b) P 2*p* regions of Ni‐DES.

Subsequently, X‐ray photoelectron spectroscopy (XPS) tests on Ni‐DES were carried out to further explore the state of substance. **Figure** **2** displays the XPS spectra of the Ni 2*p* and P 2*p* regions, indicating both Ni and P are contained in this substance. The binding energies of Ni 2*p*_3/2_ and Ni 2*p*_1/2_ are 853.93 and 871.28 eV, respectively (Figure 2a). However, for pure nickel, the binding energies of Ni 2*p*_3/2_ and Ni 2*p*_1/2_ are 852.6 and 869.9 eV, respectively.^[^ ^20^, ^21^ ^]^ Obviously, the Ni 2*p* peaks of Ni‐DES shift toward lower electron binding energy values. This shift may be due to the doping of P. When P is doped into Ni, the electron cloud of Ni atoms will shift toward P atoms, thus reducing the binding energy of Ni atoms and resulting in the shift of peak positions. The peaks at 859.26 and 878.62 eV belong to the satellite peaks of Ni 2*p*. Figure 2b presents the XPS spectra of P 2*p*. The peak at 130.03 eV can be attributed to P in P‐doped Ni metal, and the binding energy value is lower than that of elemental P (130.20 eV).^[^ ^22^ ^]^ The XPS spectra indicate that Ni‐DES is phosphorus‐doped nickel.

#### 2.1.2 Preparation of Comparative Samples

**Figure 3:**
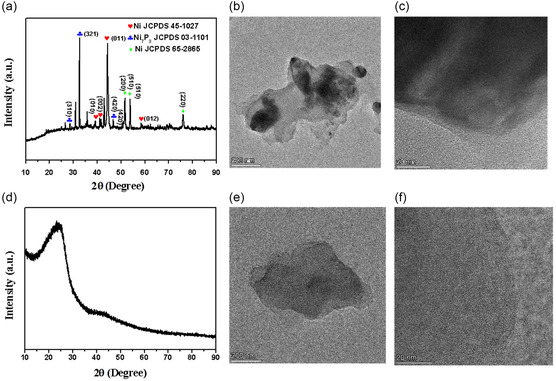
a) XRD pattern, b,c) the typical low‐ and high‐magnification TEM images of NiP‐IL; d) XRD pattern, e,f) the typical low‐ and high‐magnification TEM images of Am‐Gly.

**Scheme 1:**
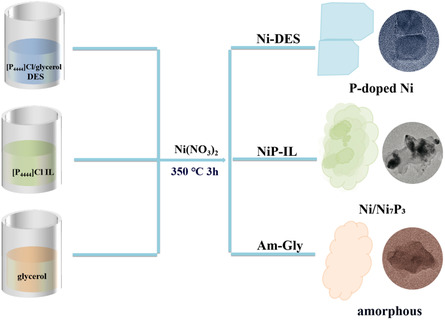
The schematic diagrams of the products obtained in [P_4444_]Cl/glycerol DES, [P_4444_]Cl IL, and glycerol.

To make a comparison with DES, we used the HBA ([P_4444_]Cl) and HBD (glycerol) of DES as media under the same conditions, respectively. After the addition of Ni(NO_3_)_2_·6H_2_O, both reactions produced black powders, abbreviated as NiP‐IL and Am‐Gly, respectively. XRD characterization revealed that the NiP‐IL obtained in [P_4444_]Cl was a mixture of Ni_7_P_3_ (JCPDS 03‐1101), Ni (JCPDS 45‐1027), and Ni (JCPDS 65‐2865) (**Figure** **3**a). TEM results showed that the obtained NiP‐IL was composed of thin sheets with uneven thickness (Figure 3b,c). When glycerol was solely used, the resulting black powder, Am‐Gly, was characterized and found to be a thin, amorphous sheet‐like substance (Figure 3d–f). The schematic diagrams of the products obtained in these three media are shown in **Scheme** **1**.

Obviously, the composition and morphology of NiP‐IL and Am‐Gly were both different from those obtained from the [P_4444_]Cl/glycerol DES. When [P_4444_]Cl is used as the solvent alone, nickel phosphide can be easily obtained. This result has also emerged in our previous work.^[^ ^23^ ^]^ That is, when the system of [P_4444_]Cl and nickel salt is microwave‐heated, nickel phosphide can also be obtained. This indicates that glycerol in DES may play the role of a reducing agent in the reaction, effectively preventing the formation of nickel phosphide. This system successfully avoids the use of potentially hazardous reducing agents such as CO and H_2_,^[^ ^24^ ^]^ greatly enhancing the safety and controllability of experimental operations. Additionally, the morphologies of the products are also different since the liquid environments provided by the three media are different. The unique solvent environment of DES may interact with Ni ions and P sources through specific interactions (such as hydrogen bonding, ion‐dipole interactions, etc.), thereby creating a specific templating effect. All in all, [P_4444_]Cl/glycerol DES plays a crucial role in the synthesis process: 1) the DES acts as a template, resulting in thin platelet with the angular; 2) DES serves as a P source; and 3) it functions as a reducing agent.

It should be noted that both white phosphorus and trioctylphosphine (TOP) can serve as phosphorus sources, but [P_4444_]Cl has unique advantages as a phosphorus source:

1. [P_4444_]Cl can form a DES with choline chloride, which is an ability that other phosphorus sources do not possess. This strategy using P‐contained DES simplifies the overall experimental conditions because the P element directly originates from the solvent itself, thereby eliminating the need for additional P sources. This reduction in complexity minimizes the possibility of impurity introduction, enhancing the purity and uniformity of the final product.
2. [P_4444_]Cl and the [P_4444_]Cl/choline chloride combination exhibit unique performance in terms of structure‐directing and templating effects. Experiments have proven that [P_4444_]Cl/choline chloride DES and [P_4444_]Cl can regulate the morphology of the catalyst, and the morphologies of the products obtained from the two solvents are different. This is beneficial for the formation of special morphologies of the phosphorus‐doped nickel catalysts, and this advantage is difficult to achieve with other phosphorus sources.
3. The DES formed by [P_4444_]Cl and choline chloride has good solubility and dispersibility, which helps the uniform distribution of reactants in the reaction system, thus playing a positive role in the synthesis of phosphorus‐doped nickel catalysts. Moreover, this DES performs excellently in terms of stability and reproducibility, providing guarantees for the stable progress of the reaction and the reproducibility of the results.
4. Other phosphorus sources, such as white phosphorus, are highly toxic substances, posing great harm to humans and the environment. Trioctylphosphine is highly corrosive and can cause severe irritation and damage to the skin, eyes, and respiratory tract. Strict protective measures are required during operation. [P_4444_]Cl belongs to ionic liquids. Ionic liquids generally have low volatility, are not easily inhaled into the human body through the respiratory tract, and have relatively low toxicity, with less irritation and corrosiveness to the human body. Meanwhile, the high stability and low volatility of [P_4444_]Cl reduce environmental pollution.

### 2.2 Performance Evaluation of Electrocatalytic Water Splitting for Hydrogen Production

**Figure 4:**
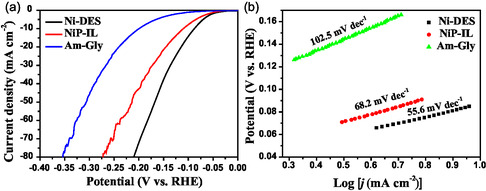
a) Polarization curves, b) Tafel plots of Ni‐DES, NiP‐IL, and Am‐Gly in 0.5 M H_2_SO_4_.

The HER performance of the obtained Ni‐DES, NiP‐IL, and Am‐Gly was tested in both acidic and alkaline conditions. In 0.5 M H_2_SO_4_, when the current density reached 10 and 20 mA cm^−2^, the overpotentials (*η*_10_ and *η*_20_) required for Ni‐DES were only 90.2 and 115.9 mV, respectively. In contrast, NiP‐IL required overpotentials of 113.6 and 146.3 mV to achieve the same current densities. For Am‐Gly, both *η*_10_ and *η*_20_ were relatively large, reaching 200.6 and 239.8 mV, respectively. In the case of high current densities, such as 50 mA cm^−2^, Ni‐DES also exhibited a small overpotential, with *η*_50_ of only 171.4 mV, while the *η*_50_ for NiP‐IL and Am‐Gly were 216.9 and 311.6 mV, respectively (**Figure** **4**a). To further explore the HER performance, we investigated the Tafel slope, which is a measure of catalytic kinetics by examining the relationship between overpotential and the logarithm of current density, as shown in Figure 4b. Ni‐DES had a small Tafel slope of 55.6 mV dec^−1^, while that of NiP‐IL and Am‐Gly were 68.2 and 102.5 mV dec^−1^, respectively.

**Figure 5:**
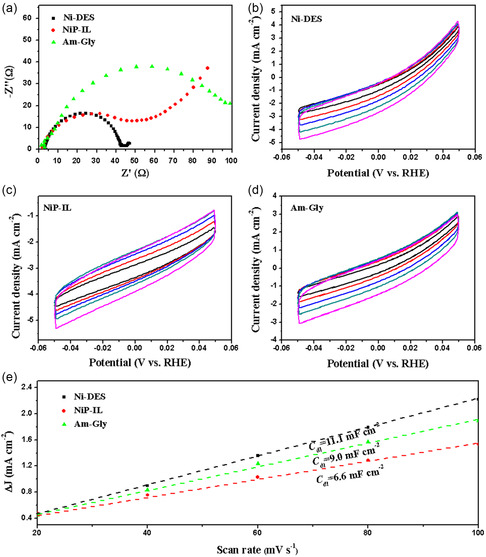
a) The EIS at 200 mV versus RHE, b) CV of Ni‐DES, c) NiP‐IL, d) Am‐Gly at scan rate of 20, 40, 60, 80, 100 mV s^−1^, e) *C*_dl_ for Ni‐DES, NiP‐IL, Am‐Gly in 0.5 M H_2_SO_4_.

The electrochemical impedance spectra (EIS) were further used to understand the electrode kinetics and interfacial properties of catalysts. As shown in **Figure** **5**a, the Nyquist plots showed the charge transfers resistance (*R*_ct_) of the obtained products. The *R*_ct_ of Ni‐DES, NiP‐IL, and Am‐Gly were 44.0, 54.2, and 107.4 Ω, respectively. These values suggested that Ni‐DES possessed the lower hydrogen adsorption impedance and faster charge‐transfer kinetics on the surface of the electrode than that of NiP‐IL and Am‐Gly, indicating that it was active for the HER. These results agreed well with those from both the overpotential and Tafel slope measurements. Furthermore, the electrochemically active surface areas (ECSA) of the studied catalysts were compared by measuring the double‐layer capacitance (*C*_dl_) (Figure 5b–d), which was in proportion to the ECSA. The *C*_dl_ of Ni‐DES was determined to be 11.1 mF cm^−2^, significantly higher than that of NiP‐IL (9.0 mF cm^−2^) and Am‐Gly (6.6 mF cm^−2^) (Figure 5e). This demonstrated that Ni‐DES could allow the more effective accessibility of active sites.

**Figure 6:**
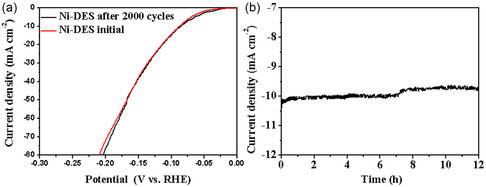
a) Polarization curves of the Ni‐DES before and after 2000 cycles, b) *I−t* curve for Ni‐DES at overpotential of 105 mV in 0.5 M H_2_SO_4_.

Besides the catalytic activity, the stability was other significant factor for evaluating an electrocatalyst. The stability test for Ni‐DES was performed in 0.5 M H_2_SO_4_ by cyclic voltammograms (CV) for 2000 cycles and the current density‐time (*I−t*) study. The polarization curve with 2000 cycles (**Figure** **6**a) showed that the linear sweep voltammetry (LSV) curves before and after 2000 cycles almost overlap. The HER performance of Ni‐DES showed no significant change after undergoing 2000 cycles. The *I−t* curve was for 12 (Figure 6b). It exhibited a slight decline in the current density near 7 h, and then it remained stable for a long time. It indicated that Ni‐DES had an excellent stability in the 0.5 M H_2_SO_4_ environment. Therefore, Ni‐DES nanocrystals could be used as an efficient electrocatalyst for the HER reaction with good long‐term cycle stability.

**Figure 7:**
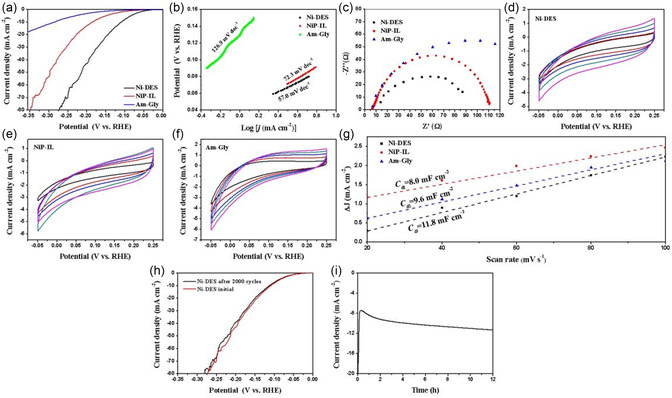
a) Polarization curves, b) Tafel plots, and c) EIS at 200 mV versus RHE of Ni‐DES, NiP‐IL, Am‐Gly. d) CV of Ni‐DES, e) NiP‐IL, and f) Am‐Gly at scan rate of 20, 40, 60, 80, and 100 mV s^−1^. g) *C*_dl_ for Ni‐DES, NiP‐IL, and Am‐Gly. h) Polarization curves of the Ni‐DES before and after 2000 cycles. i) Current density–time (*I−t*) curve for Ni‐DES at overpotential of 105 mV in 1.0 M KOH.

In 1.0 M KOH, the prepared catalyst also exhibited excellent electrocatalytic performance for HER. To achieve current densities of 10, 20, and 50 mA cm^−2^, Ni‐DES required overpotentials were 107.0, 145.4, and 214.9 mV, respectively. In contrast, NiP‐IL required overpotentials of 186.7, 226.3, and 296.3 mV to reach the same current densities. As for Am‐Gly, the *η*_10_, *η*_20_, and *η*_50_ were 295.8, 365.9, and 485.3 mV, respectively (**Figure** **7**a). Among the three catalysts, Ni‐DES exhibited the smallest Tafel slope of 57.0 mV dec^−1^, compared to 72.3 mV dec^−1^ for NiP‐IL and 126.9 mV dec^−1^ for Am‐Gly (Figure 7b). Moreover, we conducted resistance and capacitance tests on this three catalysts. The resistances of Ni‐DES, NiP‐IL, and Am‐Gly were 93.7, 111.5, and 156.7 Ω, respectively (Figure 7c). The capacitance of Ni‐DES was 11.8 mF cm^−2^, while that of NiP‐IL was 9.6 mF cm^−2^ and that of Am‐Gly was 8.0 mF cm^−2^ (Figure 7d–g). The above test prove that the synthesized P‐doped Ni offers high performance under alkaline conditions (Figure 7h,i and **Table** **1**).

**Table 1:** Comparison of HER catalytic performance of Ni‐DES, NiP‐IL, and Am‐Gly in acidic and alkaline conditions.

Catalyst	Electrolyte	Current density [mA cm^−2^]	Overpotential [mV]	Tafel slope [mV dec^−1^]
Ni‐DES	0.5 M H_2_SO_4_	10	90.2	55.6
		20	115.9	
		50	216.9	
NiP‐IL	0.5 M H_2_SO_4_	10	113.6	68.2
		20	146.3	
		50	311.6	
Am‐Gly	0.5 M H_2_SO_4_	10	200.6	102.5
		20	239.8	
Ni‐DES	1.0 M KOH	10	107.0	57.0
		20	145.4	
		50	214.9	
NiP‐IL	1.0 M KOH	10	186.7	72.3
		20	226.3	
		50	296.3	
Am‐Gly	1.0 M KOH	10	295.8	126.9

The excellent catalytic activity of Ni‐DES can be attributed to several factors. Nickel metal itself serves as a good HER catalyst due to its excellent electrical conductivity, which ensures rapid electron transfer during electrode reactions. On this basis, the doping of P can modify nickel to further improve its catalytic efficiency. The introduction of P atoms into the nickel lattice can induce lattice distortion, modulating the position of the nickel d‐band center, further optimizing the adsorption and desorption processes of hydrogen adsorption intermediates (such as H*) on the catalyst surface, thereby reducing the activation energy of HER.^[^ ^25^ ^]^ This doping effect creates favorable conditions for the efficient progress of the HER in water splitting.^[^ ^9^, ^10^ ^]^ Unique morphology is another important factor for the advanced catalytic activity of Ni‐DES. Compared to bulk materials, platelets offer additional reaction surfaces. More surfaces mean more active sites for adsorbing water molecules, enhancing HER rate. Furthermore, it allows hydrogen ions to diffuse to the active sites over shorter distances, rapidly diffusing from both sides and edges of the platelets, reducing transport resistance, and accelerating the hydrogen evolution reaction. The excellent performance is a synergistic result of composition and morphology.^[^ ^26^ ^]^

## 3 Conclusion

In this study, we have successfully synthesized P‐doped Ni materials with unique morphologies by dissolving Ni(NO_3_)_2_ in [P_4444_]Cl/glycerol DES (1:2 molar ratio) and heating at 350 C. The DES played a crucial role in the preparation process of Ni catalyst. It served as not only a reaction medium but also a P source and reducing agent. It exhibited a templating effect that induced the formation of a unique thin sheet with angular edges. The synergistic effect of this unique lamellar morphology and P doping enhances the excellent HER catalysis performance, exhibiting low overpotentials and Tafel slopes in both acidic and alkaline environments. The smart design of DES and its utilization as both a template and reactant for the preparation of transition metal catalysts provide a novel approach in the field of electrocatalyst synthesis.

## 4 Experimental Section

4.1

4.1.1

##### Materials

Tetrabutylphosphonium chloride ([P_4444_]Cl) (purity ≥ 98%) was bought from Aladdin Limited Company. Glycerol (purity: 99.5%) and nickel nitrate hexahydrate (Ni(NO_3_)_2_·6H_2_O) (purity: 99.5%) were purchased from Sinopharm Chemical Reagent Co., Ltd. Nafion solution (5 wt%) was bought from Sigma‐Aldrich. All aqueous solutions were prepared with the deionized water.

##### Preparation of DES

First, [P_4444_]Cl and glycerol were weighed according to a 1:2 molar ratio and thoroughly mixed. Then, the mixture was placed in an oil bath at 80  C and continuously stirred using a magnetic stirrer until a colorless, transparent, and homogeneous liquid was formed, which was the [P_4444_]Cl/glycerol DES.

##### Synthesis of Ni‐Based Catalyst and Comparison Samples

0.5 mmol of Ni(NO_3_)_2_·6H_2_O was added to 5 g of each of the following solvents: [P_4444_]Cl/glycerol (1:2) DES, [P_4444_]Cl and glycerol. The mixtures were then sonicated for 10 min to obtain homogeneous solutions. These solutions were transferred into crucibles and annealed in a tube furnace under N_2_ at a heating rate of 10 C min^−1^, until a temperature of 350 °C was reached and maintained for 3 h. After the reaction, the crucibles were cooled to room temperature naturally. The obtained products were washed several times with ethanol and water to remove organic impurities and byproducts, then centrifuged at 10,000 rpm to obtain black precipitates. These precipitates were dried under vacuum at room temperature for one day. To distinguish them, the obtained products were named Ni‐DES, NiP‐IL, and Am‐Gly, respectively.

##### Material Characterization

XRD characterization was conducted on a Rigaku Dmax‐rc X‐ray diffractometer. TEM characterization was examined on high‐resolution TEM, FEITecnaiF20, USA. XPS characterization was performed with Thermo Scientific ESCALAB 250Xi. Nitrogen adsorption isotherms were measured at 77 K with a Quantachrome Nova 4000 analyzer. The samples were measured after being outgassed at 423 K overnight.

##### Electrochemical Measurements

Electrochemical measurements were carried out with an electrochemical workstation (CHI model 760E) in a standard three‐electrode configuration. Ni foam modified with synthesized products served as the working electrode with a mass loading of 0.35 mg cm^−2^ modified by 0.2% Nafion. The addition of 0.2% Nafion, which is short for perfluorosulfonic acid‐based polymer, can prevent the catalyst from detaching from the Ni foam. Nafion has adhesiveness and film‐forming properties, and there is an interaction between it and the catalyst. The sulfonic acid group (—SO_3_H) contains the active center for proton conduction, which can provide a proton transport channel within the membrane, enabling protons to migrate rapidly in the membrane. A graphite rod acted as the counter electrode. A saturated calomel electrode worked as the reference electrode. All data were obtained in 0.5 M H_2_SO_4_ and 1.0 M KOH with respect to the reversible hydrogen electrode (RHE), and the iR compensation was applied. The LSV was obtained by sweeping at a potential sweep rate of 10 mV s^−1^. The EIS measurements were tested at an overpotential *η* = 200 mV (versus RHE) within the frequency range from 0.01 Hz to 100 kHz. The ECSA of Ni were obtained by cyclic voltammetry (CV), which was able to measure the double‐layer capacitance with the scanning rate ranging from 20 to 100 mV s^−1^. The stability test was conducted in 0.5 M H_2_SO_4_ and 1.0 M KOH through CV and current density‐time study. To ensure the accuracy of the experimental results, we carried out three parallel experiments.

## Conflict of Interest

The authors declare no conflict of interest.
